# What Factors Increase the Risk of Complications in SARS-CoV-2–Infected Patients? A Cohort Study in a Nationwide Israeli Health Organization

**DOI:** 10.2196/20872

**Published:** 2020-08-25

**Authors:** Chen Yanover, Barak Mizrahi, Nir Kalkstein, Karni Marcus, Pinchas Akiva, Yael Barer, Varda Shalev, Gabriel Chodick

**Affiliations:** 1 KI Research Institute Kfar Malal Israel; 2 Maccabi Institute for Research and Innovation Tel Aviv Israel; 3 Sackler Faculty of Medicine Tel-Aviv University Tel Aviv Israel

**Keywords:** SARS-CoV-2, COVID-19, risk factors, complications

## Abstract

**Background:**

Reliably identifying patients at increased risk for coronavirus disease (COVID-19) complications could guide clinical decisions, public health policies, and preparedness efforts. Multiple studies have attempted to characterize at-risk patients, using various data sources and methodologies. Most of these studies, however, explored condition-specific patient cohorts (eg, hospitalized patients) or had limited access to patients’ medical history, thus, investigating related questions and, potentially, obtaining biased results.

**Objective:**

This study aimed to identify factors associated with COVID-19 complications from the complete medical records of a nationally representative cohort of patients, with severe acute respiratory syndrome coronavirus 2 (SARS-CoV-2) infection.

**Methods:**

We studied a cohort of *all* SARS-CoV-2–positive individuals, confirmed by polymerase chain reaction testing of either nasopharyngeal or saliva samples, in a nationwide health organization (covering 2.3 million individuals) and identified those who suffered from serious complications (ie, experienced moderate or severe symptoms of COVID-19, admitted to the intensive care unit, or died). We then compared the prevalence of pre-existing conditions, extracted from electronic health records, between complicated and noncomplicated COVID-19 patient cohorts to identify the conditions that significantly increase the risk of disease complications, in various age and sex strata.

**Results:**

Of the 4353 SARS-CoV-2–positive individuals, 173 (4%) patients suffered from COVID-19 complications (all age ≥18 years). Our analysis suggests that cardiovascular and kidney diseases, obesity, and hypertension are significant risk factors for COVID-19 complications. It also indicates that depression (eg, males ≥65 years: odds ratio [OR] 2.94, 95% CI 1.55-5.58; *P*=.01) as well as cognitive and neurological disorders (eg, individuals ≥65 years old: OR 2.65, 95% CI 1.69-4.17; *P*<.001) are significant risk factors. Smoking and presence of respiratory diseases do not significantly increase the risk of complications.

**Conclusions:**

Our analysis agrees with previous studies on multiple risk factors, including hypertension and obesity. It also finds depression as well as cognitive and neurological disorders, but not smoking and respiratory diseases, to be significantly associated with COVID-19 complications. Adjusting existing risk definitions following these observations may improve their accuracy and impact the global pandemic containment and recovery efforts.

## Introduction

As of April 30, 2020, more than 3 million people worldwide contracted severe acute respiratory syndrome coronavirus 2 (SARS-CoV-2) and close to 250,000 people died of coronavirus disease (COVID-19) complications. In Israel, by that date, 16,004 individuals had been infected by the virus and 223 died from the disease. This pandemic poses grave challenges to patients, health care providers, and policy makers. Many of these challenges may be better addressed with timely stratification of patients to risk groups, based on their past and current medical characteristics. For example, reliably identifying patients at increased (or decreased) risk could guide clinical decisions (eg, hospitalization vs home care), public health policies (eg, risk-based quarantine), and preparedness efforts (eg, expected medical equipment required).

Various algorithms for identifying patients at risk for COVID-19 (severe) complications have been proposed. The Centers for Disease Control and Prevention (CDC) identified individuals 65 years and older, living in a nursing home or long-term care facility, or suffering from underlying medical conditions, particularly if not well controlled, as being at high risk for severe illness from COVID-19 [1]. Similarly, the European Centre for Disease Prevention and Control (ECDC) lists the age category >70 years and some underlying conditions as risk factors for critical illness [2]. The United Kingdom National Health Service (NHS) included solid organ transplant recipients, patients with specific cancers or severe respiratory conditions, pregnant women with significant heart disease, and those with increased risk of infection (eg, due to immunosuppressive therapies) in the highest clinical COVID-19 risk group [3]. In April 2020, approximately 1.3 million people in this group were asked to “shield” by staying at home for a period of at least 12 weeks. In addition, patients >70 years and those suffering from some underlying health conditions (eg, chronic respiratory diseases, BMI ≥40, and pregnant women) were considered in a wider vulnerable group (also referred to as the “flu group”). Finally, a more quantitative risk model (derived from Barda et al [4]) was adopted by the Israeli Ministry of Health (MoH), assigning a point for each underlying condition from a predefined list, then considering age group and point count to identify high-risk patients.

Initially, these algorithms were derived from a quickly growing number of epidemiological characterization studies (eg, [5,6]), which report the prevalence of various conditions in a population of interest, typically severe, hospitalized COVID-19 patients. These studies provide timely and important information; however, identifying risk factors calls for a comparative analysis, contrasting the prevalence of conditions in case and control populations. To date, only a handful of studies implemented such an approach, using, for example, the general population [7] or a confirmed (and symptomatic) COVID-19 patient cohort [8]. Similar to these efforts, we analyze here the medical records of all SARS-CoV-2–positive patients in a nationwide health organization (covering 2.3 million individuals). We compare the prevalence of existing conditions in complicated and noncomplicated cohorts and identify those conditions associated with COVID-19 complications in various age and sex strata. Our analysis highlights stratum-specific risk factors and may allow better identification of patients at risk in different subpopulations.

## Methods

### Data Source

Maccabi Health Services (MHS) is a nationwide health plan (payer-provider), representing a quarter of the Israeli population. The MHS database contains longitudinal data on a stable population of over 2.3 million people since 1993 (with an annual attrition rate lower than 1%). Data are automatically collected and include comprehensive laboratory data from a single central lab, full pharmacy prescription and purchase data, and extensive demographic information on each patient.

Data are available upon reasonable request. According to Israeli regulations, no patient-level secondary use medical data can be publicly shared.

### Study Design and Setting

SARS-CoV-2 polymerase chain reaction testing in Israel uses both nasopharyngeal and saliva samples. Individuals with a positive test result (as of April 22, 2020) were included in the *SARS-CoV-2–positive cohort*. Positive patients whose disease status, as updated by Israeli hospitals, deteriorated to moderate or severe (at any point in time), admitted to the intensive care unit, or died constitute the *complicated COVID-19 cohort*. Initially, the definition of disease status varied, to some extent, between hospitals but was largely based on the severity of lower respiratory tract symptoms, including pneumonia, respiratory distress, and artificial respiration, as well as shock and system failure. The remaining SARS-CoV-2–positive patients (including asymptomatic, mild COVID-19 patients, or those with unknown status) constitute the *noncomplicated COVID-19 cohort*. The follow-up period ended on April 30, 2020 (or upon patient’s death).

Patients nor the public were involved in the design, or conduct, or reporting, or dissemination plans of our research.

### Patient Characteristics

Apart from age and sex, we considered a set of existing conditions, comprising those included in the CDC, NHS, and Israeli MoH at-risk definitions, as well as a set of conditions showing significant association with flu and flu-like complications.

To identify each individual’s existing conditions, we used, when available, registries created and maintained by MHS. These registries are based on validated inclusion and exclusion criteria (considering coded diagnoses, treatments, labs, and imaging, as applicable). The registries are continuously and retrospectively (since 1998) updated based on each patient’s central medical record. Patients may be excluded from a registry when deemed misclassified by their primary physician. Linkage across registries and with other sources of information is performed via a unique national identification number. MHS registries used are: cardiovascular diseases (specifically, ischemic heart disease, congestive heart failure, peripheral vascular disease, cerebrovascular disease, and other cardiovascular diseases) [9], diabetes [10,11], hypertension [12], osteoporosis [13], chronic kidney disease [14], cognitive disorders, mental illness [15], cancer, immunosuppression (including advanced kidney disease, immunosuppressive treatment, asplenia, and organ transplant), weight disorders (obesity, overweight, and underweight), smoking, hospitalization (in the last 3 years), nursing home, and home care (home visits, home respiratory care, respiratory and feeding equipment). For other conditions, we relied on previously grouped lists of diagnosis codes (Read codes or International Classification of Diseases codes, 9th revision) [16-18]: deficiency anemia, fluid and electrolyte disorders, respiratory diseases (specifically, chronic obstructive pulmonary disease, chronic pulmonary disease, pleural effusion, aspiration pneumonia, and bronchiectasis), neurological disorders, end stage renal disease, rheumatoid arthritis, paralysis, hip fracture, lymphoma, and alcohol consumption.

### Statistical Analysis

We extracted the prevalence of the studied conditions (excluding ones with less than 20 occurrences) in the noncomplicated and complicated COVID-19 cohorts and measured the association between each condition and disease complication by computing the corresponding odds ratio (OR) and its estimated statistical significance (using Fisher exact test). We conducted the analysis separately in three age groups (18-50 years, 50-65 years, and ≥65 years), as well as four (age, sex) strata (male or female; younger or older than 65 years). Using different age groups (as sensitivity analysis) obtained similar results. Finally, to account for multiple testing, we controlled for the false discovery rate using Benjamini and Hochberg’s method [19]. All analyses were performed using version 4.0.0 of the R programming language (R Project for Statistical Computing; R Foundation). We used the STROBE (Strengthening The Reporting of OBservational Studies in Epidemiology) cohort checklist when writing our report [20].

### Ethical Approval

The study was approved by the institutional review board of MHS (0024-20-MHS).

## Results

The MHS SARS-CoV-2–positive cohort included 4353 individuals, of whom 173 deteriorated to moderate (n=87, 50%) or severe condition (n=45, 26%), were admitted to the intensive care unit (n=66, 38%, partly overlapping with other conditions), or died (n=21, 12%). This group of patients make up the complicated COVID-19 cohort. Overall, patients in the complicated COVID-19 cohort were older, suffered from more comorbidities, and were predominantly male (Table 1). Moreover, the prevalence of COVID-19 complications increased with age and more steeply for men than for women (Table 2). The risk of COVID-19 complications in men <70 years was significantly higher than in women (eg, *P*=.01 for patients 60-70 years old; see Table 2).

**Table 1:** Characteristics of the severe acute respiratory syndrome coronavirus 2 (SARS-CoV-2)–positive, complicated, and noncomplicated coronavirus disease (COVID-19) patient cohorts.

Characteristic				SARS-CoV-2 positive (n=4353)	Complicated COVID-19 (n=173)		Noncomplicated COVID-19 (n=4180)	
**Demographic information**								
	**Age (years), median (IQR)**			35 (22-54)	70 (58-80)		34 (22-52)	
		<18, n (%)		647 (15)		0 (0)		647 (15.6)
		18-50, n (%)		2354 (54.5)		21 (12.1)		2333 (56.3)
		50-60, n (%)		609 (14.1)		29 (16.8)		580 (14)
		60-70, n (%)		376 (8.7)		35 (20.2)		341 (8.2)
		70-80, n (%)		232 (5.4)		42 (24.3)		190 (4.6)
		≤80, n (%)		135 (3.1)		46 (26.6)		89 (2.1)
	Female, n (%)			1939 (44.5)	50 (28.9)		1889 (45.2)	
	Follow-up days, median (IQR)			30 (24-36)	28 (21-33)		30 (24-36)	
**Comorbidities, n (%)**								
	**Chronic respiratory diseases**			481 (11)	39 (22.5)		442 (10.6)	
		Chronic obstructive pulmonary disease		310 (7.1)		24 (13.9)		286 (6.8)
		Other chronic pulmonary disease		153 (3.5)		10 (5.8)		143 (3.4)
		Pleural effusion		41 (0.9)		4 (2.3)		37 (0.9)
	**Cardiovascular diseases**			310 (7.1)	57 (32.9)		253 (6.1)	
		Ischemic heart disease		132 (3)		27 (15.6)		105 (2.5)
		Congestive heart failure		30 (0.7)		11 (6.4)		19 (0.5)
		Cerebrovascular disease		57 (1.3)		15 (8.7)		42 (1)
		Peripheral vascular disease		23 (0.5)		7 (4)		16 (0.4)
		Other cardiovascular diseases		199 (4.6)		41 (23.7)		158 (3.8)
	Hypertension			627 (14.4)	102 (59)		525 (12.6)	
	Immunosuppression			164 (3.8)	31 (17.9)		133 (3.2)	
	Cancer			205 (4.7)	33 (19.1)		172 (4.1)	
	Deficiency anemia			423 (9.7)	18 (10.4)		405 (9.7)	
	**Liver and kidney diseases**							
		Liver disease		404 (9.3)		28 (16.2)		376 (9)
		Chronic kidney disease		384 (8.8)		86 (49.7)		298 (7.1)
		End stage renal disease		85 (2)		26 (15)		59 (1.4)
	Fluid and electrolyte disorders			394 (9.1)	37 (21.4)		357 (8.5)	
	**Metabolic diseases**							
		Diabetes		362 (8.3)		58 (33.5)		304 (7.3)
		Obesity (BMI≥30)		874 (20.1)		73 (42.2)		801 (19.2)
	**Neurological and cognitive disorders**							
		Neurological disorders		294 (6.8)		57 (32.9)		237 (5.7)
		Paralysis		53 (1.2)		12 (6.9)		41 (1)
		Depression		578 (13.3)		53 (30.6)		525 (12.6)
		Cognitive impairment		87 (2)		28 (16.2)		59 (1.4)
	**Other**							
		Hospitalization		931 (21.4)		92 (53.2)		839 (20.1)
		**Smoking**		643 (14.8)		41 (23.7)		602 (14.4)
			Current smoker	514 (11.8)		30 (17.3)		484 (11.6)
			Past smoker	129 (3)		11 (6.4)		118 (2.8)
		Nursing home		67 (1.5)		23 (13.3)		44 (1.1)
		Home care		44 (1)		17 (9.8)		27 (0.6)

**Table 2:** Association of male sex and coronavirus disease (COVID-19) complications across age groups.

Age group	Patient counts, n (%)				OR^a^ (95% CI)	*P* value^b^
	Male		Female			
	Complicated	Noncomplicated	Complicated	Noncomplicated		
18-50 years	18 (1)	1300 (99)	3 (0.3)	1033 (99.7)	4.77 (1.39-25.32)	.01
50-60 years	25 (7)	314 (93)	4 (1)	266 (99)	5.28 (1.79-21.15)	.003
60-70 years	29 (13)	202 (87)	6 (4)	139 (96)	3.32 (1.31-10.03)	.01
70-80 years	27 (20)	108 (80)	15 (15)	82 (85)	1.36 (0.65-2.95)	.47
≥80 years	24 (43)	32 (57)	22 (28)	57 (72)	1.93 (0.89-4.26)	.15

^a^OR: odds ratio. ORs greater than 1 suggest an increased risk for COVID-19 complications in males.

^b^*P* values adjusted for multiple testing using Benjamini and Hochberg’s method [19].

Comparing the prevalence of existing conditions in the three age groups between the complicated and noncomplicated COVID-19 cohorts revealed multiple risk factors, including obesity for patients 18-50 years old (OR 11.09, 95% CI 4.15-32.67; *P*<.001), chronic kidney disease for patients 50-65 years (OR 4.06, 95% CI 1.89-8.38; *P*=.005), and neurological disorders (OR 2.65, 95% CI 1.69-4.17; *P*<.001) for patients ≥65 years (for a complete list, see Table 3 and Multimedia Appendix 1).

Stratifying by age (below and above 65 years) and sex (Table 4 and Multimedia Appendix 1), we observed that kidney diseases are a risk factor in all strata (eg, OR 3.45, 95% CI 1.57-8.06; *P*=.02 in women ≥65 years). Additional risk factors included hypertension in males under 65 years (OR 4.56, 95% CI 2.35-8.55; *P*<.001); neurological disorders in females ≥65 years (OR 3.55, 95% CI 1.68-7.74; *P*=.008); cognitive impairment (OR 4.18, 95% CI 1.81-9.72; *P*=.009) and depression (OR 2.94, 95% CI 1.55-5.58; *P*=.01) in males ≥65 years. Respiratory diseases and smoking, while typically more prevalent in complicated COVID-19 patients, were not identified as significant risk factors (eg, chronic obstructive pulmonary disease in patients ≥65 years: OR 1.36, 95% CI 0.75-2.4; *P*=.63) (see Multimedia Appendix 1).

**Table 3:** Most statistically significant conditions associated with increased risk of coronavirus disease (COVID-19) complications in age-stratified patient groups.

Condition	Age group	Patient counts, n				OR^a^ (95% CI)	*P* value^b^
		With condition		Without condition			
		Complicated	Noncomplicated	Complicated	Noncomplicated		
Obesity	18-50 years	14	356	7	1977	11.09 (4.15-32.67)	<.001
Depression	18-50 years	7	229	14	2104	4.59 (1.55-12.3)	.03
Hypertension	18-50 years	4	72	17	2261	7.37 (1.76-23.41)	.04
Liver disease	18-50 years	5	125	16	2208	5.51 (1.55-16.07)	.04
Chronic kidney disease	50-65 years	14	87	27	683	4.06 (1.89-8.38)	.005
End stage renal disease	50-65 years	5	8	36	762	13.11 (3.21-48.19)	.006
Neurological disorders	≥65 years	54	113	57	317	2.65 (1.69-4.17)	<.001
Chronic kidney disease	≥65 years	70	174	41	256	2.51 (1.6-3.97)	.001
Other cardiovascular diseases	≥65 years	36	70	75	360	2.46 (1.49-4.05)	.006
Cognitive impairment	≥65 years	28	52	83	378	2.45 (1.4-4.22)	.02
Home care	≥65 years	16	22	95	408	3.12 (1.47-6.48)	.02
Hypertension	≥65 years	82	249	29	181	2.05 (1.27-3.4)	.03
Cardiovascular diseases	≥65 years	50	129	61	301	1.91 (1.22-2.99)	.03
Nursing home	≥65 years	20	35	91	395	2.48 (1.29-4.65)	.04

^a^OR: odds ratio. ORs greater than 1 suggest an increased risk for COVID-19 complications in patients with the noted condition.

^b^In each stratum, rows are sorted ascendingly by *P* value.

**Table 4:** Most statistically significant conditions associated with increased risk of COVID-19 complications in age- and sex-stratified patient groups.

Condition	Age, sex group	Patient counts					OR^a^		*P* value^b^
		With condition		Without condition					
		Complicated	Noncomplicated	Complicated	Noncomplicated				
End stage renal disease	<65 years; female	2	5	7	1370	75.7 (6.23-570.01)		.01	
Immunosuppression	<65 years; female	3	46	6	1329	14.35 (2.25-69.89)		.03	
Chronic kidney disease	<65 years; female	3	58	6	1317	11.3 (1.78-54.41)		.04	
Chronic kidney disease	<65 years; male	13	66	40	1662	8.16 (3.82-16.5)		<.001	
Hypertension	<65 years; male	17	162	36	1566	4.56 (2.35-8.55)		<.001	
Obesity	<65 years; male	25	359	28	1369	3.4 (1.88-6.14)		.001	
Hospitalization	<65 years; male	21	285	32	1443	3.32 (1.79-6.04)		.004	
End stage renal disease	<65 years; male	3	7	50	1721	14.67 (2.38-66.53)		.03	
Diabetes	<65 years; male	9	105	44	1623	3.16 (1.32-6.79)		.04	
Neurological disorders	≥65 years; female	26	66	15	136	3.55 (1.68-7.74)		.008	
Chronic kidney disease	≥65 years; female	30	89	11	113	3.45 (1.57-8.06)		.02	
Home care	≥65 years; female	10	16	31	186	3.72 (1.38-9.69)		.04	
Other cardiovascular diseases	≥65 years; female	15	33	26	169	2.94 (1.3-6.51)		.04	
Cardiovascular diseases	≥65; female	19	48	22	154	2.76 (1.29-5.85)		.045	
Cognitive impairment	≥65 years; male	16	15	54	213	4.18 (1.81-9.72)		.009	
Depression	≥65 years; male	26	38	44	190	2.94 (1.55-5.58)		.01	
Neurological disorders	≥65 years; male	28	47	42	181	2.56 (1.38-4.73)		.02	
End stage renal disease	≥65 years; male	19	26	51	202	2.88 (1.39-5.9)		.03	
Chronic kidney disease	≥65 years; male	40	85	30	143	2.24 (1.26-4.02)		.03	
Fluid and electrolyte disorders	≥65 years; male	17	22	53	206	2.99 (1.39-6.38)		.03	

^a^OR: odds ratio. ORs greater than 1 suggest an increased risk for COVID-19 complications in patients with the noted condition.

^b^In each stratum, rows are sorted ascendingly by *P* value.

## Discussion

We compared the prevalence of dozens of existing conditions in Israeli SARS-CoV-2–positive and complicated COVID-19 patient cohorts to highlight conditions associated with a high risk of complications. A few other studies have employed a similar study design to identify risk factors for COVID-19 complications. For example, Ebinger et al [8] studied a cohort of symptomatic COVID-19 individuals (N=442) and examined the association of existing conditions with disease severity; and the OpenSAFELY Collaborative explored the risk of COVID-19–related hospital death in the general population (N>17 million). We emphasize that cohort composition dictates the research question it can address: our analysis focuses on SARS-CoV-2–positive individuals, hence searches for risk factors of complications in patients who already contracted the virus (but are potentially asymptomatic), while studying the general population may combine risk factors for infection and severe COVID-19 outcome. Additionally, cohorts that consider only a subset of patients, defined based on disease outcome (eg, symptomatic or hospitalized) or otherwise nonrepresentative of the entire population (eg, demographically skewed) may introduce biases to the analysis [21]; instead, we study here all SARS-CoV-2–infected patients in a large, nationwide health organization.

Multiple studies (eg, [7,22]) have shown that COVID-19 complications are most strongly associated with age and sex. Stratifying by these factors provides readily interpretable insights on the supplemental associations (in addition to older age and male sex) between pre-existing conditions and disease complications.

Many conditions highlighted by our analysis have been previously reported [5,6,8] and are part of commonly used at-risk definitions [1,3], including hypertension, obesity, as well as kidney and cardiovascular diseases. We do, however, identify a few additional risk factors, notably depression in patients aged 18-50 years and males ≥65 years; and cognitive and neurological disorders in patients ≥65 years. These additions may be, in part, associated with the different age distribution in the ≥65 years group (median 76 years, IQR 70-83.5 years versus 72 years, IQR 68-78 years, in the complicated and noncomplicated COVID-19 cohorts, respectively) and rely on small sample size (only 7 patients aged 18-50 years with depression in the complicated COVID-19 cohort; Table 3). Nonetheless, with some preliminary support [7], they may deserve more consideration in future studies. Our analysis also points out to the reduced importance of respiratory diseases and smoking. Both conditions appear as factors in most at-risk definitions [3,5]: chronic obstructive pulmonary disease has been associated with severe COVID-19 in multiple studies [23] (though not all [6]), while the role of smoking has been somewhat controversial [23,24]. The discrepancies between our analysis and previous reports likely stem from the different cohorts analyzed: SARS-CoV-2–positive individuals, ranging from asymptomatic to severe COVID-19 versus hospitalized COVID-19 patients, respectively. Other study-related attributes (eg, country-specific characteristics) may also contribute to the varying importance of the studied risk factors.

In parallel to the COVID-19 epidemiological characterization efforts, researchers have also attempted to use retrospective observational data to derive risk models for severe COVID-19 patients [25]. Such models require ample data of COVID-19 patients for both model training and performance assessment. As such data are scarce at present, some models compromised on using data for other diseases with, supposedly, similar clinical trajectory and complications. For example, DeCapprio et al [26] trained models on US Medicare claims data to predict inpatient visits with a primary diagnosis of either pneumonia, influenza, acute bronchitis, or other specified upper respiratory infections as proxy for COVID-19 complications. However, as previously reported (eg, [27]), and in agreement with our analysis, severe COVID-19 patient characteristics differ considerably from that of other diseases, thus limiting the generalizability of such models to COVID-19 and requiring adjustments to their parameters [4].

Our study has several limitations. First and foremost, it relies on routinely maintained electronic health records, which may be inaccurate and incomplete [28]. Second, the number of complicated COVID-19 patients in the MHS data is below 200, limiting the statistical power of our analysis. Third, health care policies and, in particular, testing criteria, may systematically bias the composition of the SARS-CoV-2–positive cohort. Fourth, asymptomatic and patients with mild symptoms of COVID-19 (currently in the noncomplicated cohort) may deteriorate and eventually be part of the complicated cohort, potentially modifying the results of the analysis. Fifth, our analysis is univariate in nature, testing the association of individual conditions with COVID-19 complications; as such, it is unable to uncover more complex relations (eg, interdependencies between existing conditions and COVID-19 complications), which may be discovered by multivariate analysis. Finally, we focused on data from Israel; characteristics in other geographies may differ [27]. We attempted to mitigate some of these limitations by age and sex stratification and robust estimations of statistical significance. We also note that, at the current point in time, many of these shortcomings are shared by all published research on COVID-19.

Notwithstanding these limitations, our work adopts a novel vantage point to the problem of identifying patients at increased risk for COVID-19 complications. Importantly, as SARS-CoV-2 containment efforts focus on patients at risk for severe complications (eg, shielding vulnerable population in the United Kingdom [3]), changes in the list of considered conditions may have a substantial effect on a large number of individuals, thus calling for continuous fine-tuning of the corresponding definitions.

## Appendix

Multimedia Appendix 1 Odds ratio analysis.

## Abbreviations

CDC: Centers for Disease Control and Prevention
COVID-19: coronavirus disease
ECDC: European Centre for Disease Prevention and Control
MHS: Maccabi Health Services
MoH: Ministry of Health
NHS: National Health Service
OR: odds ratio
SARS-CoV-2: severe acute respiratory syndrome coronavirus 2
STROBE: Strengthening The Reporting of OBservational Studies in Epidemiology
